# Metacognitions associated with reproductive concerns: A cross-sectional study of young adult female cancer survivors in China

**DOI:** 10.3389/fpsyg.2022.987221

**Published:** 2022-09-27

**Authors:** Pan Pan Xiao, Si Qing Ding, Ying Long Duan, Xiao Fei Luo, Yi Zhou, Qin Qin Cheng, Xiang Yu Liu, Jian Fei Xie, Andy SK Cheng

**Affiliations:** ^1^Department of Nursing, Third Xiangya Hospital, Central South University, Changsha, China; ^2^Xiangya School of Nursing, Central South University, Changsha, China; ^3^Department of Emergency, Third Xiangya Hospital, Central South University, Changsha, China; ^4^Nethersole School of Nursing, The Chinese University of Hong Kong, Hong Kong, Hong Kong SAR, China; ^5^Hunan Cancer Hospital, Health Management Center, Changsha, China; ^6^Department of Rehabilitation Sciences, The Hong Kong Polytechnic University, Hong Kong, Hong Kong SAR, China

**Keywords:** cancer survivors, reproductive concerns, metacognitions, young adult, female

## Abstract

**Objective:**

Cancer and its treatments affect patients’ fertility potential. This study examined the prevalence of reproductive concerns and their relationship with metacognitions among Chinese young adult female cancer survivors (YAFCS).

**Methods:**

A total of 318 YAFCS (aged 18–39) completed an online survey from March to December 2021. Participants reported sociodemographic characteristics, reproductive concerns and metacognitions. Reproductive concerns were measured using the Reproductive Concerns after Cancer scale (RCAC), and metacognitions were measured by the Short Form of Metacognitions Questionnaire (MCQ-30). We used Pearson correlation analysis to examine associations between metacognitions and reproductive concerns across multiple domains and multivariable linear regression to determine the influencing factors of reproductive concerns.

**Results:**

The mean score of reproductive concern among YAFCS was 49.97 ± 12.52. A total of 57.9% of participants reported a high level of concern regarding at least one dimension of reproductive concerns, and they were most concerned about their child’s health and least concerned about partner disclosure of fertility status. We also found a moderate association between RCAC and MCQ-30 scores (*r* = 0.408, *p* < 0.001). In multivariate analyses, metacognitions, especially negative metacognitive beliefs had an impact on reproductive concerns among YAFCS (*p* < 0.001).

**Conclusion:**

Higher reproductive concerns were associated with higher metacognitions among YAFCS, especially with negative metacognitive beliefs. Oncology professionals should pay attention to assessing reproductive concerns in patients who want to have children or who have no children. Moreover, metacognitive beliefs may be an intervention target for alleviating reproductive concerns among YAFCS.

## Introduction

Cancer is the leading cause of death caused by disease in the world, and its incidence is increasing year by year and showing an obvious trend in younger people. It is estimated that the global cancer burden will be 28.4 million cases in 2040, which is a 47% rise from that in 2020 (Sung et al., 2021). Although long-term survival rates for cancer patients have improved as cancer early detection, diagnosis and treatment techniques have improved (Siegel et al., 2022), cancer-related symptoms and the secondary physiological and psychological changes caused by cancer treatment will also affect patients’ physical and mental health and lead to a decreased quality of life (Daniel et al., 2019).

Cancer and its treatments have different degrees of impairment on patients’ fertility or reproductive function, which depends on the type of treatment (Chemaitilly and Cohen, 2017). Many cancer survivors have uncertainty about their own fertility potential and/or status and face worries about the future after completing cancer treatment (Chapple et al., 2007; Britton, 2017; Jardim et al., 2021). Reproductive concerns are one of the psychosocial impacts of fertility damage on cancer survivors, especially among young adult (YA) cancer survivors or female patients. It is defined as a patient’s concerns about fertility potential, the emotional and practical barriers to achieving pregnancy, worries about one’s own physical health affecting the capacity to parent, concerns about a possible negative impact of cancer on the health of one’s offspring, and worries about disclosing possible infertility to a partner after cancer diagnosis (Carpentier et al., 2011; Gorman et al., 2014; Benedict et al., 2016a). A study conducted in Sweden showed that 58% of young adult female cancer survivors (YAFCS) reported a high level of reproductive concerns (Ljungman et al., 2018). Compared with females, YA male cancer survivors have fewer reproductive concerns, with one study reporting that 28% of male patients had reproductive concerns (Ljungman et al., 2019). Studies have shown that YA cancer survivors with clinically significant reproductive concerns are associated with emotional maladjustments (Bartolo et al., 2020a), depression (Gorman et al., 2015) and a lower quality of life (Benedict et al., 2018).

Although there is a growing number of published studies examining reproductive concerns among YAFCS (Gorman et al., 2015; Ljungman et al., 2018; Jardim et al., 2021), there is little evidence regarding a theoretical framework of reproductive concerns or potentially effective interventions. A review of the literature revealed that Wells (Wells and Matthews, 1996) proposed a metacognitions theory based on the self-regulation execution function (S-REF) model, arguing that a cognitive attentional syndrome (CAS), consisting of self-focused attention, worry and rumination, attentional bias to threat-related information, and maladaptive coping behaviors (e.g., suppression, avoidance, and minimization), contributes to anxiety, and beliefs about one’s thoughts (i.e., metacognitive beliefs) underlie the activation of CAS. Patients with a generalized anxiety disorder, for example, believe that worry is important and may impact the outcome (e.g., “if I worry, I will be prepared” or “bad thoughts can make bad things happen”) and are more likely to engage in CAS, which in turn intensifies worries and prevents more adaptive emotional processing. More interestingly, reproductive concerns, as mentioned above, consist of worry about fertility potential, personal health, and so on, and is often accompanied by excessive and inflexible monitoring for threatening signs and symptoms (e.g., ovulation, menopause, and sexual function), which seems to be consistent with the CAS. The S-REF model seems particularly applicable to reproductive concerns for several reasons. First, reproductive concern is not a specific diagnosis but rather appears to be a normal concern after a diagnosis of cancer and treatment that exists as a continuum. Second, after cancer treatment, there is uncertainty and a real chance of infertility, such that the content of the belief is not entirely irrational and less likely to be challenged (Edmondson, 2014); hence, a focus on cognitive processes rather than disputing their content could be advantageous and more acceptable to patients than approaches that test the rationality of beliefs (Baker et al., 2013).

Based on the above literature review, this study aims to (Sung et al., 2021) explore the prevalence and level of reproductive concerns in Chinese YAFCS and (Siegel et al., 2022) examine their relationship with metacognitions, the key concepts in the S-REF model, to explore the underlying mechanisms of reproductive concerns. The S-REF model emphasizes the role of negative metacognitive beliefs about thoughts and positive beliefs about the necessity to engage in worry or unhelpful styles of coping (Edmondson, 2014), and symptoms of reproductive concerns are represented within the SREF model, hence, we hypothesized that a high level of metacognitions, including positive beliefs about worry and negative metacognitive beliefs about worry, would be associated with higher reproductive concerns across all dimensions.

## Materials and methods

### Study design

This is a cross-sectional study designed to measure the reproductive concerns of young adult female cancer survivors who had completed primary treatment. The study was conducted from March to December 2021. This was an observational study. The Xiangya Nursing School of Central South University Research Ethics Committee confirmed that no ethical approval was required (E202170).

### Participants

Eligibility criteria included female patients between the ages of 18 and 39 years old with any type of cancer diagnosis who had completed the primary cancer treatment. We recruited participants who came to the hospital for regular follow-up and advertised the study in cancer groups on social media. All participants were asked to sign informed consent forms prior to participating in the online survey through WenJuanXing. A total of 434 potential participants completed the questionnaire in the above ways. We checked the data and found that 116 were ineligible. Finally, a total of 318 participants were included.

### Measurements

The survey collected self-reported information on self-designed demographic, reproductive, and cancer characteristics, reproductive concerns, and metacognitions.

#### Demographics, reproductive and cancer characteristics

Participants reported demographics, including age, race, education, job status, marriage, income, and insurance. They were also asked to report reproductive characteristics, including childbearing history, reproductive willingness, and primary caregiver during the disease (patient’s partner or others such as parents, etc.). Finally, participants reported cancer characteristics, including cancer type, metastasis status, time since completing primary treatment and cancer treatments received, including surgery, chemotherapy, radiotherapy, endocrine therapy and immunotherapy.

#### Reproductive concerns

The Reproductive Concerns after Cancer (RCAC) scale was designed by Gorman et al. (2014) and was used to assess reproductive concern among young adult female cancer patients. It has six dimensions encompassing 18 items: fertility potential, partner disclosure, child’s health, personal health, acceptance and being pregnant. Each item was rated from 1 (strongly disagree) to 5 (strongly agree), with higher scores suggesting higher levels of concern. The dimension of acceptance is reverse scored, with higher scores indicating less acceptance. A mean value of ≥ 4 in one dimension indicated a high level of reproductive concerns in that respective area (Gorman et al., 2014). The total score is the sum of the scores of all six dimensions. The Chinese version of the RCAC scale has demonstrated good reliability and validity among Chinese YAFCS, with a Cronbach’s alpha of 0.79 (Qiao et al., 2017).

#### Metacognitions

The Short Form of Metacognitions Questionnaire (MCQ-30; Cook et al., 2014) is a 30-item, validated measure that includes five subscales (six items each): positive beliefs about worry (POS), negative beliefs about worry (NEG), cognitive confidence (CC), need for control (NC) and cognitive self-consciousness (CSC). Participants rated each item on a 4-point Likert scale (1 = completely disagree, 4 = agree completely). Total scores range from 6 to 24 for each subscale, with higher scores indicating higher levels of positive and negative beliefs about worry, a belief in the need to control thoughts, a tendency toward self-focused attention, and lower levels of cognitive confidence. The Chinese version of the MCQ-30 has been validated and demonstrated good reliability and validity among Chinese university students, with a Cronbach’s alpha of 0.92 (Zhang et al., 2020). The Cronbach’s alpha of the Chinese version of the MCQ-30 in this study, including the Chinese YAFCS, was 0.94.

### Statistical analysis

All data were analyzed using SPSS 26.0 version software. The overall participant characteristics were described as frequencies and percentages for categorical variables and means and standard deviations for continuous variables. Differences between groups were examined using independent-samples t test, analysis of variance in the univariate analysis, or Chi-square test. Pearson correlation analysis was used to explore the relationship between reproductive concerns and metacognitions among YAFCS. Multivariable linear regression was used to determine the influencing factors of reproductive concerns among YAFCS. Variables with *p* < 0.2 in univariate analysis were included in this model as confounding factors. *p* < 0.05 was considered statistically significant.

## Results

### Participant characteristics

A total of 318 YAFCS were included in univariate and multivariate analyses. Summarized demographics, reproductive, and clinical characteristics are shown in Table 1. The mean age of the participants was 34.37 years old (SD = 5.46 years, ranging from 18 to 39) and mostly Han (88.7%). Most were married (80.5%), employed (83.6%), high school or technical secondary school graduates (57.9%) and had Medicare (91.8%). The top three types of cancer diagnoses were gynecological (36.5%), breast (34.6%), and thyroid cancer (16.0%). The majority of participants completed their primary treatment in less than a year (80.5%), and most of them did not have cancer metastasis (72.0%). A total of 90.6% of participants received surgical treatment, and 67.6% received chemotherapy as adjuvant treatment.

**Table 1:** Demographics, reproductive and clinical variables for young adult female cancer survivors (N = 318).

Characteristics	*N* (%)	RCAC scores	*t/F*	*p*
Age			2.261	0.024^*^
≤ 31 year ≥ 32 year	96 (30.2) 222 (69.8)	52.26 ± 13.21 48.93 ± 12.10		
Race			−0.105	0.919
Han Other	282 (88.7) 36 (11.3)	49.94 ± 12.88 50.17 ± 9.40		
Education			0.637	0.524
<College degree ≥College degree	184 (57.9) 134 (42.1)	50.35 ± 12.52 49.44 ± 13.17		
Job status			1.280	0.201
Employed Unemployed	266 (83.6) 52 (16.4)	52.00 ± 10.89 49.57 ± 12.80		
Marriage			3.568	0.021^*^
Unmarried Married Divorced	46 (14.5) 256 (80.5) 16 (5.0)	52.93 ± 15.85 49.52 ± 11.74 48.50 ± 13.65		
Income			−1.522	0.129
<5,000 ≥5,000	100 (31.4) 218 (68.6)	50.69 ± 12.25 48.39 ± 13.03		
Insurance			−0.230	0.818
Self-paying Medicare	26 (8.2) 292 (91.8)	49.42 ± 14.37 50.01 ± 12.37		
Primary caregiver			2.925	0.004^*^
Couple Other	181 (56.9) 137 (43.1)	48.20 ± 11.66 52.30 ± 13.26		
Childbearing history 0 1 ≥ 2	62 (19.5) 109 (34.3) 147 (46.2)	54.27 ± 14.79 49.29 ± 12.52 48.65 ± 11.10	4.752	0.009^*^
Reproductive willingness			−4.939	<0.001^*^
Yes None	93 (29.2) 225 (70.8)	55.17 ± 13.16 47.81 ± 11.62		
Cancer types			0.912	0.457
Gynecological cancer Breast cancer Thyroid cancer Colorectal cancer Others	116 (36.5) 110 (34.6) 51 (16.0) 11 (3.5) 30 (9.4)	50.50 ± 11.78 51.11 ± 12.07 48.25 ± 12.47 47.45 ± 16.34 47.53 ± 15.38		
Time after completing primary treatment			0.810	0.446
<1 year 1–3 year >3 year	256 (80.5) 40 (12.6) 22 (6.9)	49.56 ± 12.85 51.03 ± 11.00 52.73 ± 11.19		
Metastasis			0.103	0.918
Yes None	89 (28.0) 229 (72.0)	50.01 ± 12.10 49.84 ± 13.64		
Surgical treatment			−0.321	0.749
Yes None	288 (90.6) 30 (9.4)	50.04 ± 12.41 49.27 ± 13.79		
Adjuvant therapy				
Chemotherapy Radiotherapy Endocrine therapy Immunotherapy	215 (67.6) 93 (29.3) 57 (17.9) 10 (3.2)	50.33 ± 12.99 51.06 ± 12.44 52.95 ± 12.62 54.40 ± 10.77	−0.740 −1.006 −1.994 −1.138	0.460 0.315 0.047^*^ 0.256

* *p* value was considered statistically significant.

A total of 56.6% of participants had a spouse as their primary caregiver during their cancer survival. A total of 19.5% of participants did not have a biological child, and 29.2% of participants had reproductive willingness (wanting one or more children).

Table 2 showed the outcomes of the RCAC scale and subscale scores. The mean and standard deviation of the total score was 49.97 ± 12.52, and the scores of fertility potential, partner disclosure, child’s health, personal health, acceptance, and being pregnant were 2.59 ± 1.06, 2.35 ± 1.07, 3.30 ± 1.09, 3.30 ± 1.00, 2.43 ± 0.90, and 2.69 ± 1.00, respectively. Overall, 11.3% of participants had high concerns about fertility potential, 8.2% had high concerns about partner disclosure, 38.4% had high concerns about their (potential) child’s health, 33.0% had concerns about their own health, 8.8% had low acceptance of infertility, and 11.6% had high concerns about becoming pregnant. A total of 57.9% of participants had high concerns on at least one dimension of reproductive concerns, and 29.9% had high concerns on at least two dimensions. Table 3 showed the results of comparison between groups with different sociodemographics based on cut-off score of each subscale of the MCQ-30. fertility potential was associated with age, children number, and reproductive willingness; partner disclosure was associated with marriage, primary caregiver, children number, and reproductive willingness; personal health was associated with cancer types, chemotherapy and endocrine therapy; acceptance was associated with age, marriage, and reproductive willingness.

**Table 2:** Characteristics of Reproductive Concerns after Cancer scale (RCAC) scale and subscales scores in young adult female cancer patients.

Items	Scores	Range	Cut-off ≥ 4; *n*(%)
Fertility potential	2.59 ± 1.06	1–5	36 (11.3)
Partner disclosure	2.35 ± 1.07	1–5	26 (8.2)
Child’s health	3.30 ± 1.09	1–5	122 (38.4)
Personal health	3.30 ± 1.00	1–5	105 (33.0)
Acceptance	2.43 ± 0.90	1–5	28 (8.8)
Being pregnant	2.69 ± 1.00	1–5	37 (11.6)
RCAC Total	49.97 ± 12.52	22–86	184 (57.9)^a^ 95 (29.9)^b^

a ≥ 1 dimension above cutoff,

b ≥ 2 dimensions above cut-off. RCAC, reproductive concerns after cancer.

**Table 3:** Comparison of demographics, reproductive and clinical variables based on RCAC subscale cut-off values (*N* = 318).

Characteristics	Fertility potential		Partner disclosure		Child’s health		Personal health		Acceptance		Being pregnant	
	*χ* ^2^	*p*	*χ* ^2^	*p*	*χ* ^2^	*p*	*χ* ^2^	*p*	*χ* ^2^	*p*	*χ* ^2^	*p*
Age	3.538	0.040^*^	1.973	0.160	0.086	0.769	2.191	0.139	4.842	0.022^*^	0.004	0.948
≤ 31 year ≥32 year												
Race Han Other	0.056	0.813	0.869	0.351	1.924	0.165	2.140	0.144	2.781	0.095	0.869	0.351
Education <College degree ≥College degree	1.029	0.310	0.000	0.985	2.994	0.084	2.275	0.132	0.007	0.936	0.318	0.573
Job status Employed Unemployed	3.460	0.063	0.019	0.889	0.088	0.767	0.863	0.362	0.051	0.822	0.202	0.653
Marriage Unmarried Married Divorced	3.870	0.144	10.176	0.006^*^	0.197	0.906	2.666	0.264	7.766	0.021^*^	0.124	0.940
Income < 5,000 ≥ 5,000	1.043	0.307	0.006	0.938	0.115	0.735	0.601	0.438	0.259	0.611	0.057	0.811
Insurance Self-paying Medicare	0.371	0.542	0.009	0.925	0.000	0.992	0.379	0.538	0.044	0.834	0.387	0.534
Primary caregiver Couple Other	3.851	0.050	3.934	0.047^*^	1.605	0.205	0.443	0.506	1.378	0.240	1.168	0.280
Childbearing history 0 1 ≥ 2	14.776	0.001^*^	12.820	0.002^*^	5.854	0.054	3.029	0.220	5.233	0.073	1.214	0.545
Reproductive willingness Yes None	19.921	<0.001^*^	14.270	<0.001^*^	2.568	0.109	0.115	0.735	4.381	0.036^*^	1.494	0.222
Cancer types Gynecological cancer Breast cancer Thyroid cancer Colorectal cancer Others	1.900	0.754	0.898	0.925	5.968	0.202	21.004	<0.001^*^	1.903	0.754	3.008	0.556
Time after completing primary treatment < 1 year 1–3 year > 3 year	1.102	0.576	0.564	0.754	0.083	0.956	1.879	0.519	3.060	0.217	3.352	0.187
Metastasis Yes None	1.470	0.225	0.016	0.900	0.981	0.322	4.089	0.053	0.136	0.712	0.279	0.598
Surgical treatment Yes None	0.004	0.950	0.101	0.751	0.355	0.552	1.405	0.236	0.059	0.808	0.351	0.553
Adjuvant-therapy Chemo- Radio- Endocrine- Immune-	0.016 0.034 0.510 0.018	0.898 0.854 0.475 0.893	0.477 0.395 0.124 0.046	0.490 0.530 0.725 0.831	3.430 0.709 2.381 0.049	0.064 0.400 0.123 0.824	12.743 3.654 8.143 2.256	<0.001^*^ 0.056 0.004^*^ 0.133	0.155 0.621 2.366 0.018	0.694 0.431 0.124 0.892	0.144 1.494 0.389 0.048	0.704 0.222 0.533 0.901

* *p < 0.05.*

The association between metacognitions and demographics, reproductive, and clinical characteristics was analyzed. Cancer types (*F* = 2.620, *p* = 0.035) and endocrine treatment (*t* = −2.246, *p* = 0.025) were found to be related to metacognitions in YAFCS.

### Association between reproductive concerns and metacognitions

A Pearson correlation analysis showed a moderate association between RCAC and MCQ-30 total score (*r* = 0.408, *p* < 0.001). Except for the correlation between acceptance and NEG, CC, CSC and MCQ-30 total score, which did not have statistical significance, the relationships between other variables were all statistically significant (*r* = 0.112 ~ 0.442, *p* < 0.05; Table 4). Besides, independent samples t test showed that compared with participants without clinically significant reproductive concerns, participants with clinically significant reproductive concerns had higher scores of MCQ-30, POS, NEG, CC, NC, and CSC (*p* < 0.05; Table 5).

**Table 4:** Correlations between reproductive concerns and metacognitions in young adult female cancer survivors.

Items	POS	NEG	CC	NC	CSC	MCQ-30 total
Fertility potential	0.262^**^	0.324^**^	0.134^*^	0.245^**^	0.172^**^	0.284^**^
Partner disclosure	0.311^**^	0.341^**^	0.186^**^	0.287^**^	0.209^**^	0.333^**^
Child’s health	0.122^*^	0.274^**^	0.112^*^	0.200^**^	0.241^**^	0.241^**^
Personal health	0.141^*^	0.381^**^	0.305^**^	0.272^**^	0.275^**^	0.351^**^
Acceptance	0.148^**^	0.085	−0.010	0.112^*^	−0.055	0.070
Being pregnant	0.270^**^	0.377^**^	0.246^**^	0.311^**^	0.252^**^	0.366^**^
RCAC total	0.309^**^	0.442^**^	0.241^**^	0.352^**^	0.275^**^	0.408^**^

* *p* < 0.05;

** *p* < 0.01. POS, positive beliefs about worry; NEG, negative beliefs about worry; CC, cognitive confidence; NC, need for control; CSC, cognitive self-consciousness.

**Table 5:** Comparison of metacognitions between clinically significant and non-clinical significant reproductive concerns in young adult female cancer survivors.

Variables	Clinical significant reproductive concerns		*t*	*p*
	No	Yes		
MCQ-30	60.42 ± 15.55	67.90 ± 16.46	−4.094	<0.001^*^
POS	10.25 ± 3.56	11.35 ± 3.99	−2.839	0.012^*^
NEG	12.21 ± 4.44	14.53 ± 4.42	−4.606	<0.001^*^
CC	12.22 ± 4.36	14.14 ± 4.50	−2.808	0.017^*^
NC	11.54 ± 3.82	13.08 ± 4.18	−3.355	0.001^*^
CSC	14.34 ± 3.52	15.80 ± 3.67	−3.554	<0.001^*^

* *p* < 0.05.

### Factors influencing reproductive concerns among young adult female cancer survivors

In the multiple linear regression model, adjusted with covariates, including age, marriage, monthly income, primary caregiver, endocrine therapy, childbearing history and reproductive willingness, MCQ-30, POS, NEG, CC, CSC, and NC were, respectively, included in the model as independent variables, results showed that MCQ-30, POS, NEG, CC, CSC, and NC were influencing factors of reproductive concerns, and NEG had the greatest impact on reproductive concerns among YAFCS (Table 6).

**Table 6:** Multivariable linear model for reproductive concerns measured by RCAC for young adult female cancer survivors.

Variable	Mean (SD)	*b*	SE	Standardized *b*	*t*	*p*
MCQ-30 total	64.75 (16.479)	0.311	0.037	0.409	8.353	<0.001
POS	10.89 (3.846)	0.971	0.169	0.298	5.748	<0.001
NEG	13.55 (4.567)	1.196	0.132	0.436	9.052	<0.001
CC	12.75 (4.458)	0.764	0.148	0.272	5.181	<0.001
NC	12.43 (4.098)	1.060	0.154	0.347	6.881	<0.001
CSC	15.19 (3.672)	0.971	0.178	0.285	5.449	<0.001

POS: *R*^2^ = 0.183, *F* = 9.939, *p* < 0.001; NEG: *R*^2^ = 0.285, *F* = 17.670, *p* < 0.001; CC: *R*^2^ = 0.168, *F* = 8.961, *p* < 0.001; NC: *R*^2^ = 0.216, *F* = 12.203, *p* < 0.001; CSC: *R*^2^ = 0.183, *F* = 9.939, *p* < 0.001; MCQ-30: *R*^2^ = 0.262, *F* = 15.746, *p* < 0.001.

SD, standard deviation; SE, standard errors.

## Discussion

This study explored the level of reproductive concerns after cancer and among YAFCS and their associations with metacognitions. The RCAC score of these participants was 49.97 ± 12.52, and 57.9% of participants reported high concerns on at least one dimension of reproductive concerns, which is consistent with a study conducted by Ljungman and colleagues in Sweden (Ljungman et al., 2018). To our knowledge, this study is the first to examine the relationships between metacognitions of YAFCS and reproductive concerns. As with our hypothesis, metacognitions and their dimensions were moderately associated with reproductive concerns among YAFCS, i.e., patients with higher level of reproductive concerns had higher level of metacognitions and its five dimensions. In addition, we found that metacognitions had the greatest impact on reproductive concerns among YAFCS, indicating that interventions targeting dysfunctional metacognitive beliefs need to be designed to alleviate reproductive concerns among YAFCS in the future.

In this study, we identified a meaning level of concerns on each dimension (a mean score of 4 or greater) according to Gorman and colleagues’ study (Gorman et al., 2014) and found that YAFCS were most concerned about their child’s health (3.30 ± 1.09, 38.4%), followed by concerns about their own health (3.30 ± 1.00, 33.0%), and least concerned about partner disclosure of fertility status (2.35 ± 1.07, 8.2%), which is similar to the results of previous studies (Benedict et al., 2016b; Ljungman et al., 2018). In this study, 71% of YAFCS were concerned about passing on a genetic risk for cancer to their (potential) children and affecting children’s health, independent of hereditary cancer diagnosis. Some cancer treatments, such as endocrine and chemotherapy, have been proven to have fetotoxicity (Benedict et al., 2017). Additionally, more than half of the participants in this study were diagnosed with gynecological or breast cancer that is strongly associated with fertility, which leads them to fear that being pregnant might cause cancer recurrence (Ljungman et al., 2018). Only 8.2% of females in this study were concerned about partner disclosure of fertility status. However, there are some studies showing that many cancer survivors feared being abandoned or rejected by (potential) partners once they disclosed their reproductive failure (Armuand et al., 2015; Ussher and Perz, 2019), especially with younger patients (Gorman et al., 2014). In the studies including YA male cancer survivors (Ljungman et al., 2019; Drizin et al., 2021), the child’s health remains the greatest concern for male survivors, while personal health was less of a concern. This may be explained by the fact that the process of giving birth to a child takes place inside a woman, and hormonal changes during pregnancy could be linked to the development of cancer, such as breast, gynecological and thyroid cancer. Therefore, oncology professionals should provide counseling on the risks related to cancer genetics for both males and females who want to have child(ren). In particular, young women with hormone-dependent cancers need to be provided with cancer treatment options and appropriate timing and precautions for being pregnant, as well as periodic assessments of cancer and fetal progress to alleviate distress caused by reproductive concerns.

Our studies also showed that participants whose primary caregivers were spouses had lower levels of reproductive concerns than those whose primary caregivers were others, such as parents and siblings. This result indicated that patients’ partners play an important role in the development of reproductive concerns in YAFCS. Studies have shown that potential fertility impairment caused by cancer or its treatment not only leads to higher levels of reproductive concerns in patients but also affects their partners’ expectations about their role in biological parenthood, thus leading to psychological responses similar to those of patients (Manne and Badr, 2008; Gietel-Habets et al., 2018). Moreover, facing the reproductive concerns brought on by cancer, spouses’ coping style has an indirect impact on patients’ emotions, and their supportive behavior has a potential protective or positive effect that cannot be replaced by other relationships (Coyne and DeLongis, 1986; Casu et al., 2019). Future research should apply reliable tools to measure dyadic coping patterns between cancer patients and their partners and explore the relationship with reproductive concerns to direct intervention research for alleviating concerns.

Interestingly, we found that metacognitions and its dimension were associated with reproductive concerns among YAFCS, and metacognitions was an important influencing factor of reproductive concerns. This might be explained by the fact that potential fertility risk after cancer induces patients to have normal worries about fertility and related dimensions. However, with external stimuli such as abnormal menstruation in the survival period, individuals’ negative beliefs about worry are activated, and their level of concern is aggravated, which is often accompanied by negative emotions such as distress, anxiety or depression (Cook et al., 2015; Fisher et al., 2015; Ng et al., 2020). Most YA female breast cancer survivors in many previous studies reported that their worry about their children’s health or their personal health during pregnancy persists, no matter how they try to stop them (Partridge et al., 2004; Ellis et al., 2016). A quarter of YAFCS who had high reproductive concerns in a study by Gorman and colleagues reported moderate to severe depression (Gorman et al., 2015); more importantly, concerns were reported to be associated with a lower quality of life of cancer patients (Bartolo et al., 2020b). Therefore, psychosocial interventions are urgently needed for YAFCS with high levels of reproductive concerns, and the results of this study suggest that dysfunctional metacognitive beliefs might be a potential intervention mechanism. As with reproductive concerns, fear of recurrence (FCR) is a worry that arises after cancer diagnosis and treatment, which affects patients’ psychology and quality of life (Fardell et al., 2016). Recently, an RCT study examining the effect of a psychosocial intervention that incorporates metacognitive therapy (MCT) on FCR demonstrated that this intervention could reduce FCR-related distress and cancer-specific distress (Butow et al., 2017). MCT has been considered a novel, transdiagnostic approach to treat mental disorders, in which emotional disorders are attributed to the defect in controlling the negative emotions of negative thoughts and beliefs about the concerns and the lack of using effective methods to counteract negative emotions. Dysfunctional metacognitive beliefs might be a relevant factor associated with the process of adapting to illness, and MCT aims to reduce the dysfunctional metacognitive beliefs (Normann and Morina, 2018). What is more, the metacognitive models for MCT are usually slightly different for different types of mood disorders. In order to construct a complete metacognitive model about reproductive concerns in YAFCS, it is necessary to explore the trigger of reproductive concerns in YAFCS, as well as maladaptive behaviors, negative emotions and the specific manifestations of worries in patients with persistent high levels of reproductive concerns. According to this, future research should construct a metacognitive model specific to reproductive concerns after cancer. Our hypothetical metacognitive model of high level reproductive concerns is that, cancer survivors experienced low-level or normal reproductive concerns when specific triggers are existed and positive metacognitive beliefs are activated, this anxiety persists and increases after patients’ negative cognitive beliefs are activated, and then leads to CAS related to reproductive concerns, which in turn strengthens dysfunctional metacognitive beliefs, finally forming a vicious cycle. Regarding the CAS related to reproductive concerns, how much time patients spend dwelling on or worrying about the fertility-related problems and focusing attention on the things patients find threatening, and what behaviors did patients do to deal with negative thoughts or feelings should be measured to come up with a complete metacognitive model about reproductive concerns. What is more, it is of great importance for oncology professionals to explore the effect of MCT based on the specific model on reproductive concerns among YAFCS.

### Limitations

There were some limitations to this study. Importantly, reproductive willingness was regarded as a dichotomous variable, and research tools tested for reliability and validity, such as the Fertility Intention Scale (Li et al., 2018), were not applied to measure it. Secondly, its cross-sectional nature does not allow us to determine the mechanisms of metacognitions on reproductive concerns in YAFCS. Future research employing longitudinal designs would illuminate whether reproductive concerns of YAFCS are largely caused by dysfunctional metacognitive beliefs. Thirdly, this study used convenience sampling as a recruitment strategy that could cause selection bias because those who show interest and self-select to participate in a study may be more interested in the subject matter. Further research on the reproductive concerns of YAFCS might utilize alternative recruitment methods, such as population-based cancer registries, to minimize this bias. Finally, this study did not investigate information on the psychiatric history, comorbid medical disease and psychotropic drugs history that might affect measurement results of participants’ reproductive concerns after cancer and metacognitions.

## Conclusion

In this study, 57.9% of participants reported high concerns about at least one dimension of reproductive concerns, and they were most concerned about their child’s health and least concerned about partner disclosure of fertility status. Oncology professionals should pay attention to assessing reproductive concerns in patients who want to have children or who have no children. Additionally, dysfunctional metacognitive beliefs may be an intervention target for alleviating reproductive concerns among YAFCS.

## Data availability statement

The raw data supporting the conclusions of this article will be made available by the authors, without undue reservation.

## Ethics statement

The studies involving human participants were reviewed and approved by E202170. The patients/participants provided their written informed consent to participate in this study.

## Author contributions

PX, XFL, and YZ performed material preparation, data collection, and analysis. PX, SD, and JX wrote the first draft of the manuscript and all authors commented on previous versions of the manuscript. All authors approved the final manuscript, and contributed to the study conception and design. All authors contributed to the article and approved the submitted version.

## Funding

This study was funded by The National Natural Science Foundation of China (82073409).

## Conflict of interest

The authors declare that the research was conducted in the absence of any commercial or financial relationships that could be construed as a potential conflict of interest.

## Publisher’s note

All claims expressed in this article are solely those of the authors and do not necessarily represent those of their affiliated organizations, or those of the publisher, the editors and the reviewers. Any product that may be evaluated in this article, or claim that may be made by its manufacturer, is not guaranteed or endorsed by the publisher.
